# Characterization of ACE2 naturally occurring missense variants: impact on subcellular localization and trafficking

**DOI:** 10.1186/s40246-022-00411-1

**Published:** 2022-09-02

**Authors:** Sally Badawi, Feda E. Mohamed, Nesreen R. Alkhofash, Anne John, Amanat Ali, Bassam R. Ali

**Affiliations:** 1 Department of Genetics and Genomics, College of Medicine and Health Sciences, United Arab Emirates University, P.O. Box: 15551, Al-Ain, United Arab Emirates; 2 Zayed Centre for Health Sciences, United Arab Emirates University, Al-Ain, United Arab Emirates

**Keywords:** Angiotensin-converting enzyme 2 (ACE2), Subcellular trafficking, SARS-CoV-2, Polymorphism

## Abstract

**Background:**

Human angiotensin-converting enzyme 2 (ACE2), a type I transmembrane receptor physiologically acting as a carboxypeptidase enzyme within the renin-angiotensin system (RAS), is a critical mediator of infection by several severe acute respiratory syndrome (SARS) corona viruses. For instance, it has been demonstrated that ACE2 is the primary receptor for the SARS-CoV-2 entry to many human cells through binding to the viral spike S protein. Consequently, genetic variability in ACE2 gene has been suggested to contribute to the variable clinical manifestations in COVID-19. Many of those genetic variations result in missense variants within the amino acid sequence of ACE2. The potential effects of those variations on binding to the spike protein have been speculated and, in some cases, demonstrated experimentally. However, their effects on ACE2 protein folding, trafficking and subcellular targeting have not been established.

**Results:**

In this study we aimed to examine the potential effects of 28 missense variants (V801G, D785N, R768W, I753T, L731F, L731I, I727V, N720D, R710H, R708W, S692P, E668K, V658I, N638S, A627V, F592L, G575V, A501T, I468V, M383I, G173S, N159S, N149S, D38E, N33D, K26R, I21T, and S19P) distributed across the ACE2 receptor domains on its subcellular trafficking and targeting through combinatorial approach involving in silico analysis and experimental subcellular localization analysis. Our data show that none of the studied missense variants (including 3 variants predicted to be deleterious R768W, G575V, and G173S) has a significant effect on ACE2 intracellular trafficking and subcellular targeting to the plasma membrane.

**Conclusion:**

Although the selected missense variants display no significant change in ACE2 trafficking and subcellular localization, this does not rule out their effect on viral susceptibility and severity. Further studies are required to investigate the effect of ACE2 variants on its expression, binding, and internalization which might explain the variable clinical manifestations associated with the infection.

**Supplementary Information:**

The online version contains supplementary material available at 10.1186/s40246-022-00411-1.

## Introduction

The global infection and mortality rates of the Coronavirus Disease 2019 (COVID-19), caused by the novel severe acute respiratory syndrome coronavirus 2 (SARS-CoV-2), continue to increase and thus inflecting unprecedent economic and health burden worldwide [1]. The highly contagious SARS-CoV-2 displays a wide spectrum of clinical presentations ranging from asymptomatic infection to severe cardiorespiratory failure that acquire hospitalization, mechanical respiratory, and cardiac support for some cases [2]. Understanding the basis of the extreme interindividual clinical variability may aid clinicians and researchers to assign the most suitable and personalized supportive treatments to patients, improve the efficacy of the available vaccines, and potentially aid in the development of novel effective therapies. Unhealthy habits like smoking or individuals affected by chronic conditions such as obesity, hypertension, cardiovascular diseases, and diabetes are more prone to severe illness and worse prognosis [3, 4]. In addition to the viral genome variability, other risk factors may influence disease clinical severity and presentation including patient’s gender, race, age, and his/her genetic make-up [5].

Multiple studies have linked genetic variations in angiotensin-converting enzyme 2 (ACE2) to the clinical heterogeneity in infected patients as the receptor is primarily utilized via SARS-CoV-2 for cellular entry to initiate the infection process [6]. Various studies have investigated the influence of naturally occurring single nucleotide polymorphisms (SNPs) on infection variable susceptibility and severity through its effect on binding affinity to the viral S protein targeting ACE2 for COVID-19 therapy [6, 7]. For example, it has been speculated that the East Asian populations would be more susceptible to the severe form of the infection due to certain high allele frequency variants that may lead to higher ACE2 expression [8]. However, multiple ACE2 variants were found to exert protective effects against COVID-19 through impairing ACE2 expression and/or function [9, 10]. ACE2 is a metalloproteinase type 1 transmembrane protein made of 805 amino acids and mainly plays a role in balancing the renin-angiotensin system (RAS) through the conversion of angiotensin II (Ang II) to angiotensin 1–7 [1]. ACE2 receptor is trafficked to the cell surface through the secretory pathway where it is initially synthesized in the endoplasmic reticulum (ER). Once properly folded and post-translationally modified, ACE2 is transported to the Golgi apparatus for further complex posttranslational modifications and folding and then transported to the plasma membrane by vesicular transport [1]. In the ER, tagged misfolded proteins are directed for proteasomal degradation via the endoplasmic reticulum associated degradation (ERAD) [11].

By the beginning of the spread of COVID-19 infection in 2019, Cao et al. and other groups have demonstrated that ACE2 expression levels and genetic variation may influence its interaction with the SARS-CoV-2 spike (S) protein [8, 12, 13]. Therefore, the highlighted variable outcomes may explain some of the interindividual variability of the infection onset, susceptibility, and severity [9]. Different genetic variants were accounted to mainly affect ACE2 binding affinity to the viral S protein, its expression level, or internalization but little is known regarding its effect on the receptor processing and trafficking through the secretory pathway to the plasma membrane [6]. Some missense variants in secretory proteins like plasma membrane proteins and receptors are known for their deleterious effects and disease causation at various levels [14, 15]. Amino acid substitutions lying away from critically and functionally important protein domains may indirectly result in a loss of function effect due to total or partial retention of the protein in the ER and thus mis-trafficking [14, 16–18]. Despite their possible intact biological function, mis-localized membranous proteins lose their function due to their quantitative or partial loss from their distinct functional cellular location. In fact, the Q1069R missense mutation in the ACE2 homologue, angiotensin-converting enzyme (ACE), was found to be sequestered by the ER quality control machinery and prevented from trafficking to the cell surface [19]. Therefore, we hypothesize that some missense variants in ACE2 receptor might exert trafficking defects on this receptor and its levels at the plasma membrane. Moreover, considering ACE2 critical biological functions, partial ER retention or delay might potentially explain some of the interindividual COVID-19 clinical variability and may provide a drug target for SARS-CoV-2 and other coronaviruses infections [1, 19].

**Fig. 1:**
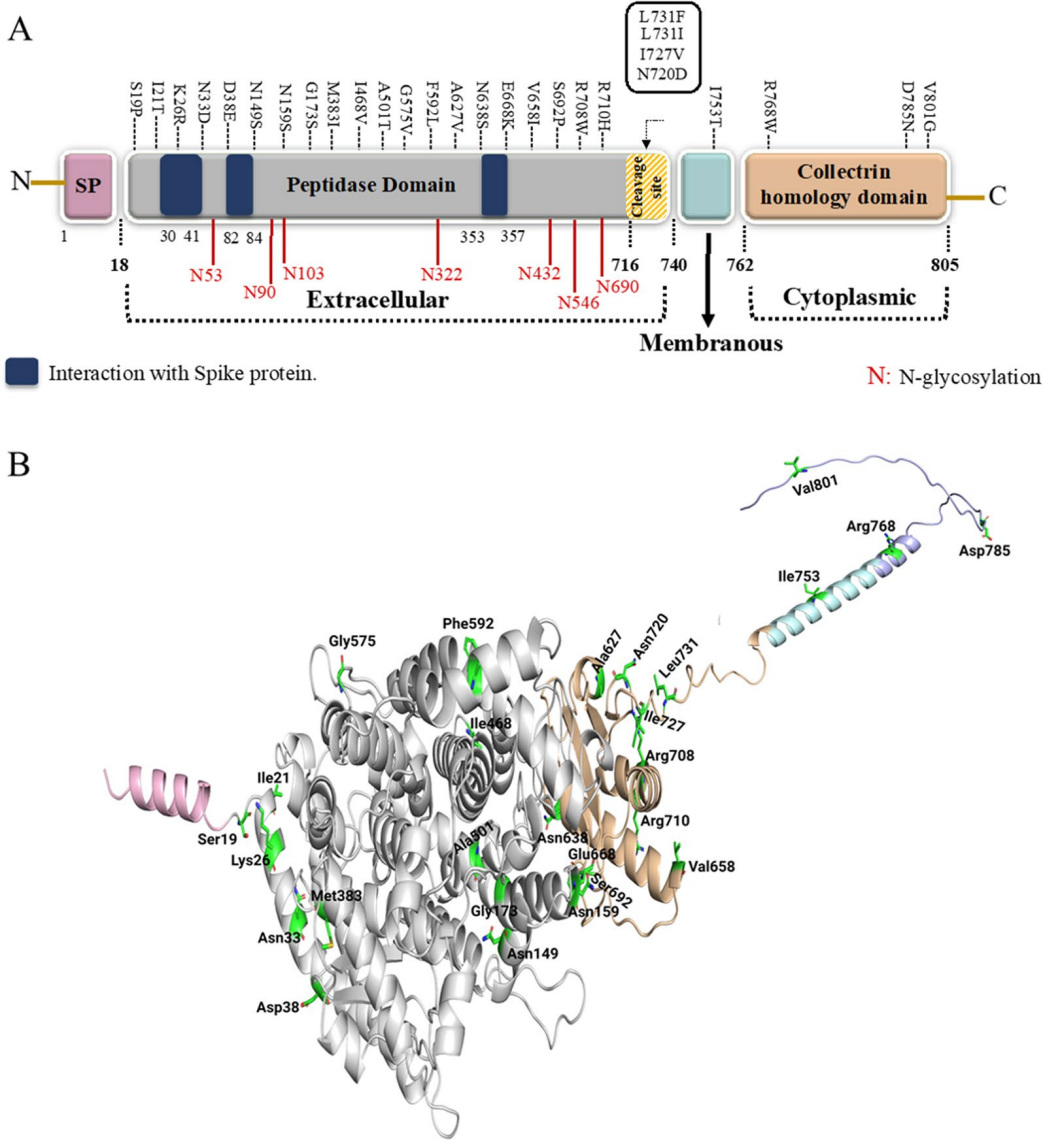
ACE2 protein domains, coding missense variants, and full-length three-dimensional structure. **A** A schematic diagram displaying the different domains of human ACE2 including the signal peptide domain (SP), peptidase domain and collectrin homology domain. Dark blue regions correspond to the residues interacting with SARS-CoV-2 S protein. Residues labeled in red correspond to the 7N-glycosylation sites in ACE2. The studied coding variants are labeled in black and distributed based on their domain. **B** 3D structure of ACE2 protein along with its functional domains. Twenty-seven positions are only shown where 2 variants are available in Leu731 position. Signal peptide (pink), peptidase domain (gray), collectrin domain (orange), transmembrane domain (cyan), and cytoplasmic domain (purple) are shown in cartoon representation and amino acids are shown in stick representation

In their comparative genetic analysis at the time of initiating this study, Cao et al. have pointed out 30 missense variants out of total 62 genetic variations in the ACE2 coding region for their potential effect on the protein amino acid sequence (Fig. 1A) [8]. We decided then to generate all those variants and evaluate their effects on the subcellular localization and N-glycosylation profile of the ACE2 receptor. Our findings indicate very limited or no detectable effects of these variations on the subcellular localization of ACE2 which may augment the notion that the biological functions of this receptor are essential, and its partial loss cannot be tolerated.

## Methodology

### In silico prediction of the structural effects of ACE2 variants

ACE2 reference SNP cluster ID (rsID) with their global allele frequency was retrieved from *Ensembl* database (https://asia.ensembl.org/Homo_sapiens/Transcript/Variation_Transcript/Table?db=core;g=ENSG00000130234;r=X:15494566-15607236;t=ENST00000427411). To assess the effect of 28 ACE2 nonsynonymous variants on protein function, different in silico prediction tools have been used. SIFT (Sorting Intolerant from Tolerant) algorithm (https://sift.bii.a-star.edu.sg) was utilized to predict whether the studied ACE2 variants affect ACE2 protein function. If SIFT’s score < 0.05 the variant is considered tolerated, and if score > 0.05 the variant is considered to affect protein function [20]. PolyPhen-2 (Polymorphism Phenotyping v2) algorithm (http://genetics.bwh.harvard.edu/pph2/index.shtml) was also used to predict the impact of ACE2 missense variants on its structure and function using different sequence and structure-based predictive features. Two pairs of datasets, HumanDiv and HumanVar, were used to evaluate the consequent damage. PolyPhen-2 scores range between 0 and 1. Benign variants have scores in the range of 0 and 0.15, possibly damaging variants have a score in the range of 0.15 and 0.9 and confidently damaging variants have a score between 0.9 and 1 [21]. PROVEAN (Protein Variation Effect Analyzer) software (http://provean.jcvi.org/seq_submit.php) was also used to predict whether ACE2 amino acid substitutions influence its biological function [22], where a score less than − 2.5 correspond to a deleterious variant. Finally, to evaluate whether these variants might be disease causing, Mutation Taster has been applied (https://www.mutationtaster.org/MutationTaster69/index.html) [23]. If a variant was found to affect protein function in at least one of the prediction tools, it is classified as possibly deleterious, otherwise it is benign.

### Analysis of the effect of missense variants on protein stability and their impact on protein structure using protein modeling

I-Mutant tool was employed to predict the effect of selected missense variants on the protein stability [24]. For this, the protein sequence of ACE2 and missense variants data was submitted in FASTA format. The primary amino acid sequence of ACE2 obtained from the UniProt (Accession No. Q9BYF1) and 28 missense variants generated manually were used as input. Moreover, the coordinates of ACE2 crystal structure were obtained from the protein data bank (PDB), PDB ID 6M17, excluding the coordinates of receptor binding domain (RBD) of SARS-CoV-2 from the co-complexed B^o^AT1 dimer. I-TASSER was used to model the missing C terminal residues (769–805) and N terminal residues (1–19) of the ACE2 transmembrane helices [25]. SWISS-MODEL was used to generate the three-dimensional homology models of mutant proteins using modeled full-length structure of ACE2 as a template [26].

### Mutagenesis primers design and generation of ACE2 missense variants by site-directed mutagenesis

FLAG-tagged Human ACE2 wild type plasmid (NM_021804) was purchased from OriGene Inc. (RC208442). Among Cao et al.’s reported variants, we have generated 28 missense variants using the wild type construct as a template, via Quick-Change site-directed mutagenesis kit with the *Pfu* Ultra High-Fidelity DNA polymerase (Stratagene). The primers used for the mutagenesis were designed (Additional file 1: Table S1) using PrimerX software (https://www.bioinformatics.org/primerx/) and purchased from Metabion International AG (https://www.metabion.com/). The generation of the desired variants was confirmed by the dideoxy Sanger DNA sequencing using the ABI 3130xl automated fluorescent Genetic Analyzer (Applied Biosystems). Clustal Omega software (https://www.ebi.ac.uk/Tools/msa/clustalo/) was used for sequence alignments.

### Cell culture

HeLa and HEK293 cells were cultured in Dulbecco’s Modified Eagle Medium (Gibco) supplemented with 10% fetal bovine serum (Gibco), antibiotic–antimycotic (Gibco) at 37 °C and 5% CO2 as previously described [27].

### Immunofluorescence and confocal microscopy

HeLa cells were seeded on sterilized cover slips for imaging. Cells were co-transfected with WT or mutated ACE2 plasmid and GFP-tagged HRas, a plasma membrane marker. The methodology has been described previously [27]. Twenty-four hours later, cells were washed three times with phosphate-buffered saline (PBS) and then fixed with methanol at − 20 °C for 5 min. Fixed cells were then blocked with 3% bovine serum albumin (Sigma-Aldrich) for 30 min at room temperature. Fixed cells were co-stained with Anti-Flag primary antibody (1:100 Cell Signaling) and anti-Calnexin (1:50 Santa Cruz Biotechnology) for 1 Hrs in the dark at room temperature. Cells were then washed three times with PBS and then incubated with the respective secondary antibody (Thermo Fischer Scientific) for 45 min in the dark at room temperature. Afterward, cells were then washed and mounted with immmunofluor medium (ICN Biomedicals) and images were acquired using the 100 × objective Nikon confocal Eclipse 80I microscope (Nikon Instruments Inc.). Images were further analyzed and merged using ImageJ software [28].

### SDS-PAGE immunoblotting

Forty-eight hours post transfection, HEK293 cells seeded in 6 well-plates were lysed according to manufacturers’ instructions in RIPA lysis buffer (Pierce Inc.) along with protease inhibitor cocktail (Pierce). Total proteins were quantified by the colorimetric bicinchoninic acid protein assay (BCA kit, Pierce). 20 µg total protein lysate was resolved on 4–12% SDS-PAGE precast gradient gel (GeneScript) followed by transfer into PVDF membrane. Membrane was then incubated with primary antibodies: anti-ACE2 (1:1000 Santa Cruz, cat# sc-390851) anti-Flag antibody (1:1000 Cell Signaling, cat # 8146S), anti-GFP (1:1000 Cell Signaling, cat# 2955S) and anti-actin (1:1000 Santa Cruz Biotechnology, cat# sc-47778) and their corresponding secondary antibodies (Sigma-Aldrich). Membranes were then incubated with Enhanced Chemiluminescence Plus reagent (Pierce) and developed using the Typhoon FLA 9500 imager (GE Healthcare Biosciences, Piscataway, NJ, USA). Blot analysis quantification was then performed using ImageJ software [28].

### Glycosylation sensitivity and resistance assays

HEK293 cells seeded in 6-well plates were co-transfected with wild type or mutant ACE2 and GFP plasmids. Forty-eight hours later, cells were harvested, and lysates were then denatured in denaturation buffer at 100 °C for 10 min based on manufacturer’s protocol. Equal amounts of the proteins were incubated at 37 °C for 3 Hrs in presence or absence of 10U of endoglycosidase H (Sigma-Aldrich). Samples were then resolved on 4–12% SDS-PAGE gel and processed for western blotting as previously described.

N-linked oligosaccharides were removed by PNGase F treatment (New England Biolabs). Cell lysates were denatured at 100 °C for 10 min and equal amounts of glycoproteins were then incubated at 37 °C for 1 Hrs in presence and absence of PNGase F enzyme. Samples were then resolved on 4–12% SDS-PAGE gradient gels and proceeded for western blotting.

### Protein stability analysis and half-life determination

Twenty-four hours post transfection, HEK293 cells seeded in 6 well-plates were treated with 100 μg/ml cycloheximide (CHX) (Sigma-Aldrich) to stop new protein translation for different time intervals (4, 8, 12, 18, and 24 Hrs). DMSO-treated cells at the same time intervals were taken as control. Cells were then harvested and proceeded by western blotting.

## Results

### In silico analysis of ACE2 naturally occurring missense variants

All 28 ACE2 missense variants were tested using different bioinformatic predictive tools to identify the possible functional effects of these variants on ACE2. Altogether, only 3 variants (R768W, G575V, and G173S) were predicted to be deleterious by all the evaluated algorithms (Table 1). Twelve variants were detected to affect protein function by SIFT algorithm. Only 4 variants were found to be deleterious by PROVEAN analysis and 13 were predicted to be damaging by PolyPhen-2 HumanVar, and PolyPhen-2 HumanDiv. Predictions by Mutation Taster show that 10 of the studied substitutions might be disease causing where all others have no predicted phenotypic effect and are considered polymorphic. In total, 10 variants (V801G, N720D, E668K, V658I, F592L, N159S, N149S, D38E, K26R, and I21T) were found to have no effect on the function of ACE2 by all the tested algorithms, classified as benign (Additional file 1: Table S2).

**Table 1:** ACE2 missense coding variants predictions by different computational tools

ACE2 mutant	SNP	AA substitution		SIFT		PROVEAN		HumanDiv PolyPhen.2		HumanVar PolyPhen.2		Mutation taster
			gnomAD allele frequency	Effect	Score	Prediction	Score	Prediction	Score	Prediction	Score	Prediction
c.2353G>A	rs373153165	D785N	3.867e− 05	APF	0.00	Neu	− 0.416	Ben	0.102	Ben	0.001	Pol
c.2302C>T	rs140016715	R768W	1.385e−05	APF	0.00	Del	− 2.822	Dam	1	Pro Dam	0.996	DC
c.2258T>C	NA	I753T	NA	APF	0.01	Neu	− 0.763	Dam	0.887	Dam	0.62	Pol
c.2191C>T	rs147311723	L731F	0.001	APF	0.00	Neu	− 1.124	Pro Dam	0.975	Dam	0.695	DC
c.2191C>A	NA	L731I	NA	APF	0.00	Neu	− 0.669	Ben	0.443	Dam	0.45	Pol
c.2179A>G	NA	I727V	NA	APF	0.00	Neu	− 0.421	Ben	0.011	Ben	0.044	Pol
c.2129G>A	rs370187012	R710H	3.912e−05	APF	0.00	Neu	− 1.788	Pro Dam	1	Pro Dam	1	DC
c.2122C>T	rs776995986	R708W	6.918e−06	APF	0.00	Del	− 3.105	Pro Dam	1	Pro Dam	0.998	Pol
c.2074 T>C	rs149039346	S692P	0.0003776	APF	0.02	Neu	− 1.260	Dam	0.678	Dam	0.578	Pol
c.1913A>G	rs183135788	N638S	0.0002628	APF	0.03	Neu	− 1.242	Ben	0.226	Ben	0.041	Pol
c.1880C>T	rs748163894	A627V	1.095e−05	Tol	0.09	Neu	− 1.532	Dam	0.888	Ben	0.279	DC
c.1724G>T	NA	G575V	NA	APF	0.00	Del	− 8.358	Dam	1	Dam	0.984	DC
c.1501G>A	rs140473595	A501T	1.259e−05	Tol	0.09	Neu	− 2.025	Dam	0.858	Ben	0.235	DC
c.1402A>G	rs191860450	I468V	0.001	Tol	0.44	Neu	− 0.339	Pro Dam	0.966	Dam	0.793	DC
c.1149G>A	NA	M383I	NA	Tol	0.11	Neu	− 2.431	Pro Dam	0.992	Pro Dam	0.957	DC
c.517G>A	rs754511501	G173S	2.182e−05	APF	0.02	Del	− 5.891	Pro Dam	0.962	Dam	0.82	DC
c.97A>G	NA	N33D	NA	Tol	0.34	Neu	− 1.225	Ben	0.054	Ben	0.059	DC
c.55 T>C	rs73635825	S19P	0.0002518	Tol	0.18	Neu	− 0.757	Dam	0.767	Dam	0.74	Pol

Tol, tolerated; APF, affect protein function; Neu, neutral; Del, deleterious; Ben, benign; Dam, possibly damaging; Pro Dam, probably damaging; Pol, polymorphism; DC, disease causing; NA, not available

### Analysis of the structural stability and effect of missense variants on ACE2 protein

I-mutant Suite was performed to evaluate the effect of missense variants on the overall protein stability. Results demonstrated that all studied variants decreased the ACE2 stability, compared to wild type ACE2 except for S19P, D38Q and S692P variants (Table 2). Among the 28 studied variants, I21T, N33D, F592L, D785N, and V801G were found to introduce the highest instability in ACE2 with a Gibbs free energy change value (ΔΔG) of − 2.16, − 2.10, − 2.97, − 2.16, and − 3.20, respectively.

**Table 2:** ACE2 variants stability profile and energy calculations

ACE2 mutant	SNP	AA substitution	Stability	RI (0–10)	ΔΔG (Kcal/mol)
c.2402T>G	rs1464340051	V801G	Decrease	9	− 3.20
c.2353G>A	rs373153165	D785N	Decrease	8	− 2.16
c.2302C>T	rs140016715	R768W	Decrease	6	− 1.01
c.2258T>C	rs931448406	I753T	Decrease	4	− 1.24
c.2191C>T	rs147311723	L731F	Decrease	6	− 0.35
c.2191C>A	NA	L731I	Decrease	5	− 0.55
c.2179A>G	NA	I727V	Decrease	6	− 0.66
c.2158A>G	rs41303171	N720D	Decrease	7	− 1.38
c.2129G>A	rs370187012	R710H	Decrease	8	− 1.85
c.2122C>T	rs776995986	R708W	Decrease	7	− 1.21
c.2074T>C	rs149039346	S692P	Increase	1	1.41
c.2002G>A	rs200180615	E668K	Decrease	8	− 1.10
c.1972G>A	rs1295899858	V658I	Decrease	5	− 0.37
c.1913A>G	rs183135788	N638S	Decrease	8	− 1.11
c.1880C>T	rs748163894	A627V	Decrease	5	− 0.86
c.1774T>C	NA	F592L	Decrease	8	− 2.97
c.1724G>T	NA	G575V	Decrease	3	− 0.13
c.1501G>A	rs140473595	A501T	Decrease	5	− 0.96
c.1402A>G	rs191860450	I468V	Decrease	8	− 0.70
c.1149G>A	NA	M383I	Decrease	2	− 0.02
c.517G>A	rs754511501	G173S	Decrease	7	− 0.48
c.476A>G	rs746034076	N159S	Decrease	6	− 0.47
c.446A>G	rs373252182	N149S	Decrease	4	− 0.40
c.114C>G	NA	D38E	Increase	3	0.02
c.97A>G	NA	N33D	Decrease	4	− 2.10
c.77A>G	rs4646116	K26R	Decrease	5	− 0.34
c.62T>C	rs1244687367	I21T	Decrease	8	− 2.16
c.55T>C	rs73635825	S19P	Increase	6	0.39

SNP, single nucleotide polymorphism; AA, amino acid; RI, Reference Index; ΔΔG, Gibbs free energy

**Fig. 2:**
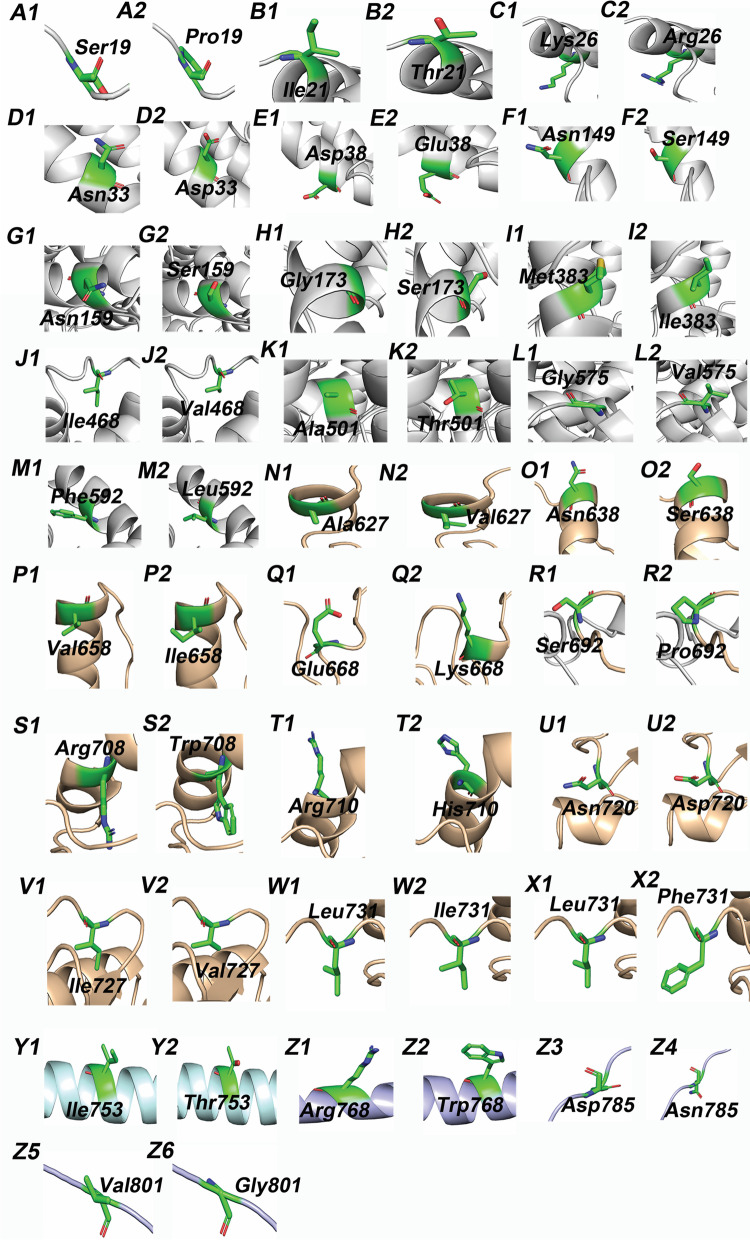
The enlarged view of the residues surrounding missense variants shown in Fig. 1B. **A1** wild type Ser19; **A2** mutant Pro19; **B1**wild type Ile21; **B2** mutant Thr21; **C1** wild type Lys26; **C2** mutant Arg26; **D1** wild type Asn33; **D2** mutant Asp33; **E1** wild type Asp38; **E2** mutant Glu38; **F1** wild type Asn149; **F2** mutant Ser149; **G1** wild type Asn159; **G2** mutant Ser159; **H1** wild type Gly173; **H2** mutant Ser173; **I1** wild type Met383; **I2** mutant Ile383; **J1** wild type Ile468; **J2** mutant Val468; **K1** wild type Ala501; **K2** mutant Thr501; **L1** wild type Gly575; **L2** mutant Val575; **M1** wild type Phe592; **M2** mutant Leu592; **N1** wild type Ala627; **N2** mutant Val627; **O1** wild type Asn638; **O2** mutant Ser638; **P1** wild type Val658; **P2** mutant Ile658; **Q1** wild type Glu668; **Q2** mutant Lys668; **R1** wild type Ser692; **R2** mutant Pro692; **S1** wild type Arg708; **S2** mutant Trp708; **T1** wild type Arg710; **T2** mutant His710; **U1** wild type Asn720; **U2** mutant Asp720; **V1** wild type Ile727; **V2** mutant Val727; **W1** wild type Leu731; **W2** mutant Ile731; **X1** wild type Leu731; **X2** mutant Phe731; **Y1** wild type Ile753; **Y2** mutant Thr753; **Z1** wild type Arg768; **Z2** mutant Trp768; **Z3** wild type Asp785; **Z4** mutant Asn785; **Z5** wild type Val801; and **Z6** mutant Gly801. Signal peptide (pink), peptidase domain (gray), collectrin domain (orange), transmembrane domain (cyan), and cytoplasmic domain (purple) are shown in cartoon representation and amino acids are shown in stick representation

Moreover, protein modeling was performed to determine the effect of missense variants on ACE2 structure and function. The full-length ACE2 protein was modeled, and missense variants were generated on the protein structure (Fig. 1B). Among 28 studied variants, 13 are located in peptidase domain (PD) of ACE2 which is a potential binding site for RBD of the S protein of SARS-CoV-2. Although some variants are present distant from the ACE2 interface and do not form direct interactions with the RBD of SARS-CoV-2, they might exert structural deformities on ACE2 protein. The evaluation of S19P indicated that the substitution of conserved serine into a hydrophobic proline is likely to disturb the interactions with other ACE2 residues important for protein structure (Fig. 2A1, A2). The substitution of non-polar isoleucine to a polar threonine at 21 amino acid position is likely to produce additional hydrogen bonds with A25 and E87 residues of ACE2 which could disrupt protein conformation (Fig. 2B1, B2). Moreover, the physiochemical changes of K26R and N33D variants are significant and have been shown to enhance the binding affinity of SARS-CoV-2 (Fig. 2C1–D2). However, the substitution of charged residues at amino acid position 38 is less likely to cause significant changes in protein conformation and SARS-CoV-2 binding affinity (Fig. 2E1, E2). Asparagine for serine substitutions at positions 149 and 159 are physiochemically insignificant and may have no effect on protein structure or function (Fig. 2F1–G2). G173 is buried in the core of ACE2 and is also important in forming the catalytic site. G173S variant is likely to disrupt the catalytic site formation as well as catalysis and substrate specificity (Fig. 2H1, H2). The physiochemical properties of M383I, I468V and G575l variants are insignificant and are unlikely to disrupt protein structure and function (Fig. 2I1–L2). A501T variant is expected to disturb the polar interaction of alanine with Glu181 (Fig. 2K1, K2).

Out of 11 variants present in the collectrin domain, five variants (A627V, N638S, V658I, I727V, and L731I) are physiochemically insignificant and are not observed to affect protein structure and function (Fig. 2N1–W2). E668K variant is likely to disrupt the polar interaction of wild type glutamic acid with E667 which could be important for protein conformation (Fig. 2Q1, Q2). The substitution of polar serine with a hydrophobic proline residue at 692 position is likely to disrupt the hydrogen bonds of serine with N159 and N690 (Fig. 2R1, R2). The substitution of charged arginine with neutral tryptophan at 708 position is expected to disrupt the hydrogen bonds and salt bridges formed by R708 with D719, L722, E723, and I727 residues of ACE2 (Additional file 2: Fig. S1, S2). Such changes of S692P and R708W could lead to improper protein folding. Residues 636–658 and 708–717 present at the neck region are observed to form stable polar interactions. These interactions are important for stable dimer formation. The possible loss of charge in case of R710H variant is likely to disturb these interactions, which could result in improper dimer formation (Fig. 2T1, T2). I753T, R763W, D785N, and V801G variants are located at the cytoplasmic end of ACE2 (Fig. 2Y1–Z6). The substitutions are physiochemically significant, particularly R763W, but given their location and the lack of obvious intramolecular interactions, these variants are unlikely to affect protein structure or function.

### Exogenously expressed FLAG-tagged wild type ACE2 localizes to the plasma membrane and has a half-life of about 12 Hrs

ACE2 endogenous protein expression in the human HEK293 and HeLa cell lines was assessed by immunoblotting assay using anti-ACE2 monoclonal antibody (Santa Cruz; 1:1000 dilution, cat# sc-390851). Our blot shows that ACE2 protein was not detectable in either HEK293 or HeLa cell lines even when high amounts of cell lysates (50 μg) were analyzed (Additional file 2: Fig. S1A). However, overexpressed Flag-tagged ACE2 in HEK293 cells was detected with the same anti-ACE2 specific antibody at ~ 120 KDa molecular weight.

**Fig. 3:**
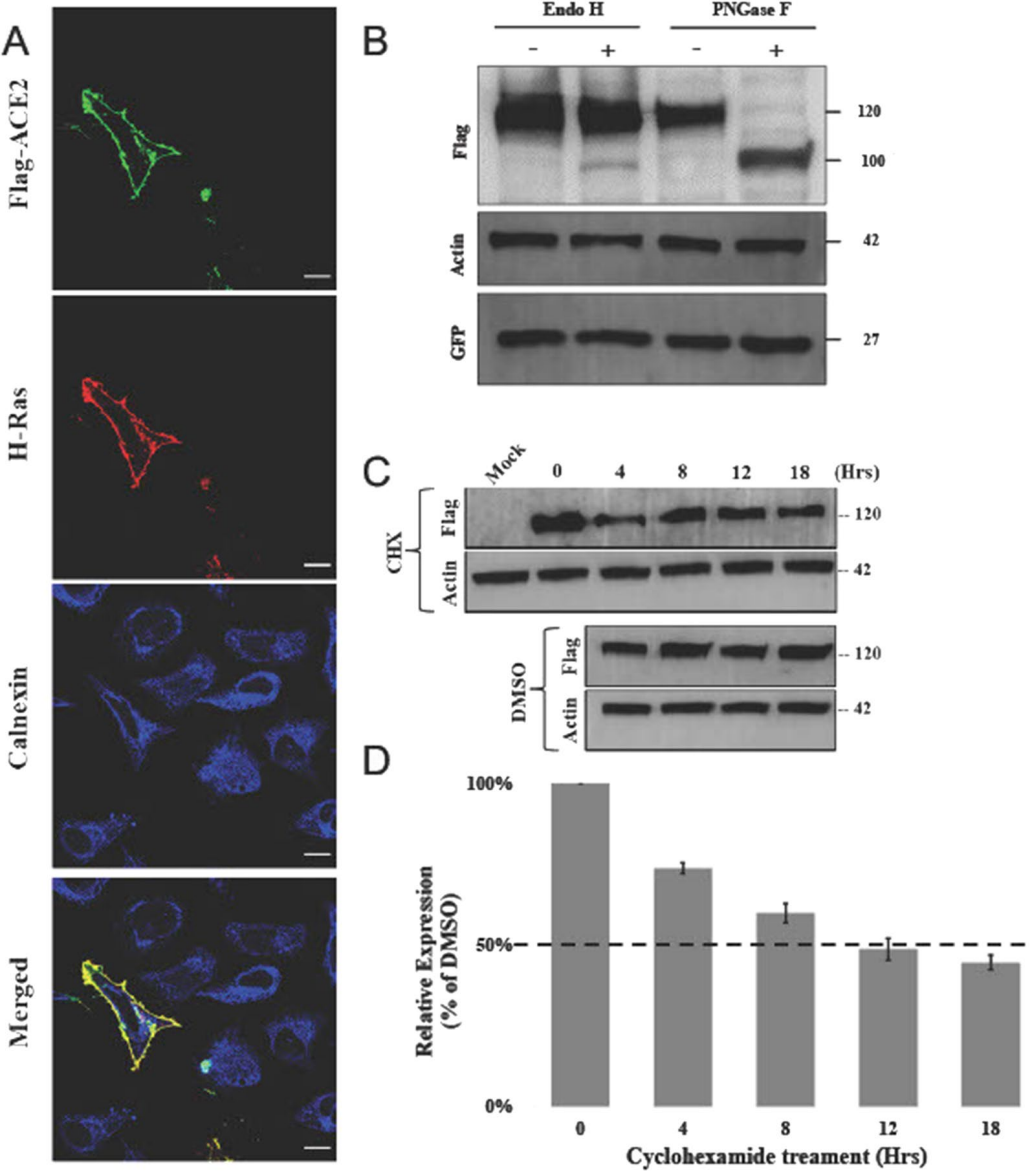
Overexpressed WT ACE2 subcellular localization and stability. **A** Immunofluorescence confocal imaging of permeabilized HeLa cells transfected with Flag-tagged ACE2 (red), GFP-tagged HRas (plasma membrane marker in green) and Calnexin (ER marker in blue). Images were acquired using 100X magnification and manipulated by ImageJ. Scale bar = 50 μm. **B** HEK293 cells transiently transfected with GFP and Flag-tagged WT ACE2 plasmids for 48  Hrs. Lysates were then digested in presence or absence of Endo H and PNGase F enzymes for 3 Hrs and 1  Hrs, respectively. Anti-Flag primary antibody was used to stain WT ACE2 and anti-GFP antibody was used to satin GFP that was used as a transfection control. **C** Flag-tagged WT ACE2 transfected cells were treated with DMSO and 100 μg/ml cycloheximide for a period of 18 Hrs. Cells were lysed at the indicated time points (0, 4, 8, 12, and 18 Hrs) and lysates were analyzed by western blotting. Anti-Flag antibody was used to stain WT ACE2. Mock sample represent un-transfected cells. In all the experiments actin was used as a loading control. **D** Graph representing the relative expression of WT ACE2 compared to DMSO treated cells at the indicated time points. Error bars represent ± SEM of three independent experiments. Band quantification was performed using ImageJ

To establish the intracellular localization of wild type overexpressed ACE2, HeLa cells were co-transfected with Flag-tagged ACE2 and GFP-tagged HRas plasmids. Confocal microscopy images of stained cells displayed a plasma membranal profile for WT ACE2 which overlapped with GFP-tagged HRas as shown in Fig. 3A. Red staining of WT ACE2 by anti-Flag antibody, colocalizes with the green GFP-tagged HRas, staining the plasma membrane, where no colocalization with the blue stained ER marker, Calnexin, was displayed. Similarly, overexpressed ACE2 displays a similar pattern in HEK293 (Additional file 2: Fig. S1B). To confirm the trafficking of ACE2 across the secretory pathway, the N-glycosylation profile of WT ACE2 was evaluated. Glycoprotein N-glycosylation is an enzyme directed process that occurs at specific asparagine residue in N-X-T/S sequence motif, in the ER that is then further modified in the Golgi apparatus. ACE2 is reported to have 7N-glycosylation sites as shown in Fig. 1A [29, 30]. Digestion of protein lysates from HEK293 cells overexpressing WT ACE2 with Endoglycosidase H (Endo H) enzyme, which cleaves off the immature N-glycans only, showed that around 2.8% ± 0.36 of the protein is digestible to a lower molecular weight band (~ 100 KDa) suggestion high level of maturation of the WT protein. The remaining 97.2% did not change their molecular weight protein band of ~ 120KDa suggesting that it has acquired the complex N-glycans that usually take place in the Golgi complex before trafficking to the plasma membrane (Fig. 3B). On the other hand, treatment of N-glycans of ACE2 WT protein by the PNGase F enzyme, a peptide N-glycosidase F that cleaves off N-glycans regardless of their glycans maturation stage, resulted in shift from a high molecular weight band (120 KDa) to a lower molecular weight protein band (~ 100KDa). These results demonstrate that WT ACE2 acquires fully mature N-glycans quantitatively and presumably has high maturation rate.

To gain insight on the half-life, stability, and turnover of the overexpressed WT ACE2, we overexpressed the protein for 24 Hrs then added the protein synthesis inhibitor cycloheximide at 100 µg/ml and quantified the remaining ACE2 by Western blotting at several subsequent time points up to 18 Hrs. As shown in Fig. 3C, D, overexpressed WT ACE2 has an approximate half-life of 12 Hrs suggesting a relatively slow turn over.

### All studied ACE2 variants traffic normally and localize to the plasma membrane resembling the WT protein

**Fig. 4:**
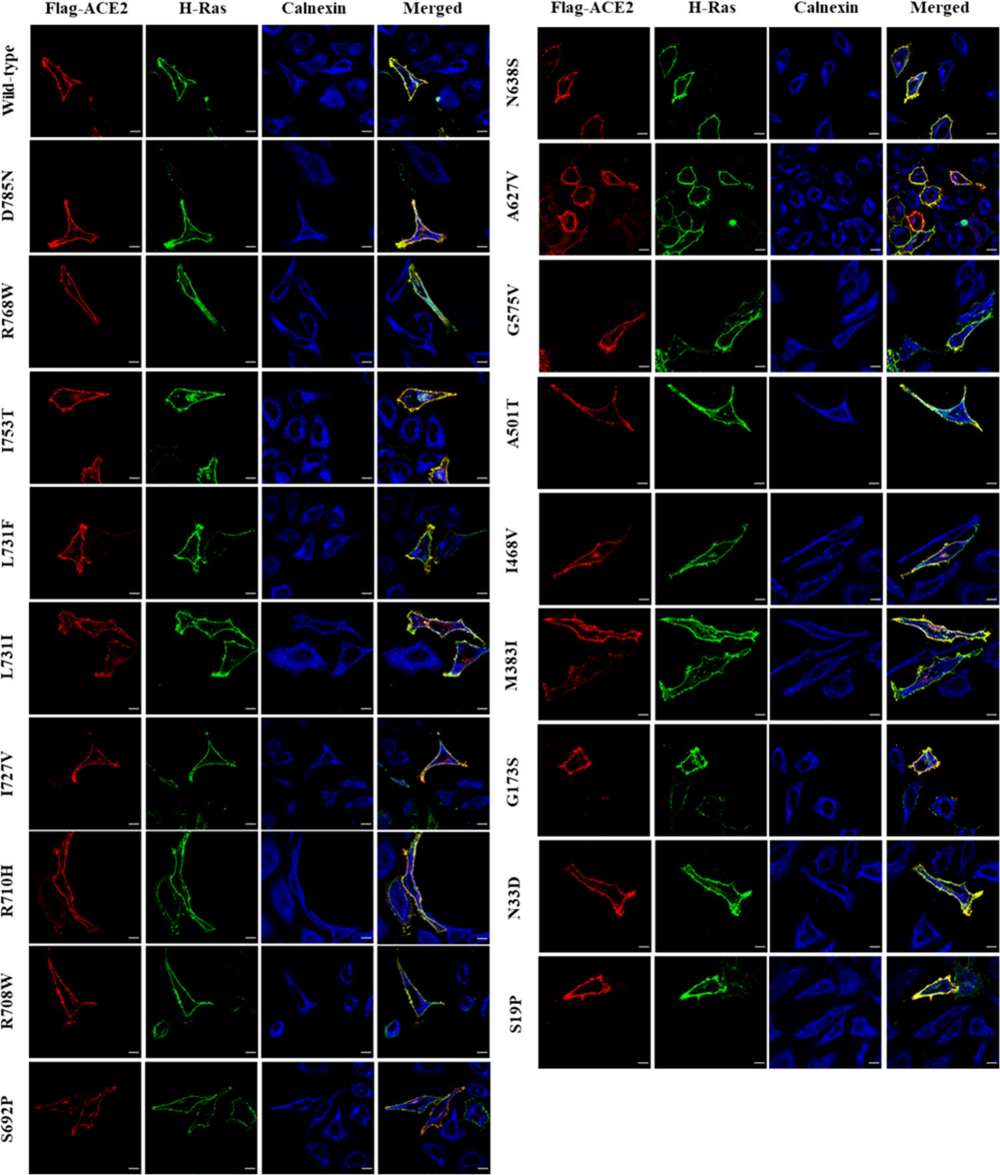
ACE2 variants subcellular localization. HeLa cells were grown on coverslips and transiently co-transfected with Flag-tagged ACE2 WT or missense variants and GFP-tagged HRas (plasma membrane marker). 24 Hrs post transfection, cells were fixed and stained with anti-Flag and anti-Calnexin (ER marker) antibodies. Images were acquired using 100X magnification and manipulated by ImageJ. Scale bar = 50 μm

To assess the effects of the studied ACE2 missense variants, we expressed them individually in HeLa and HEK293 cell lines. HEK293 cells overexpressing ACE2 mutants display normal protein expression pattern by western blotting compared to WT ACE2 (Additional file 2: Fig. S2). HeLa cells were co-transfected with an ACE2 construct (WT or mutants) and GFP-tagged HRas plasmids then the subcellular localization of the proteins was evaluated by confocal microscopy. Interestingly, as shown in Fig. 4 and in Additional file 2: Fig. S3, none of these variants (including the possibly deleterious variants) affected the apparent subcellular localization of the ACE2 protein. For all the studied variants, ACE2 appeared at the plasma membrane and co-localized with GFP-tagged HRas.

**Fig. 5:**
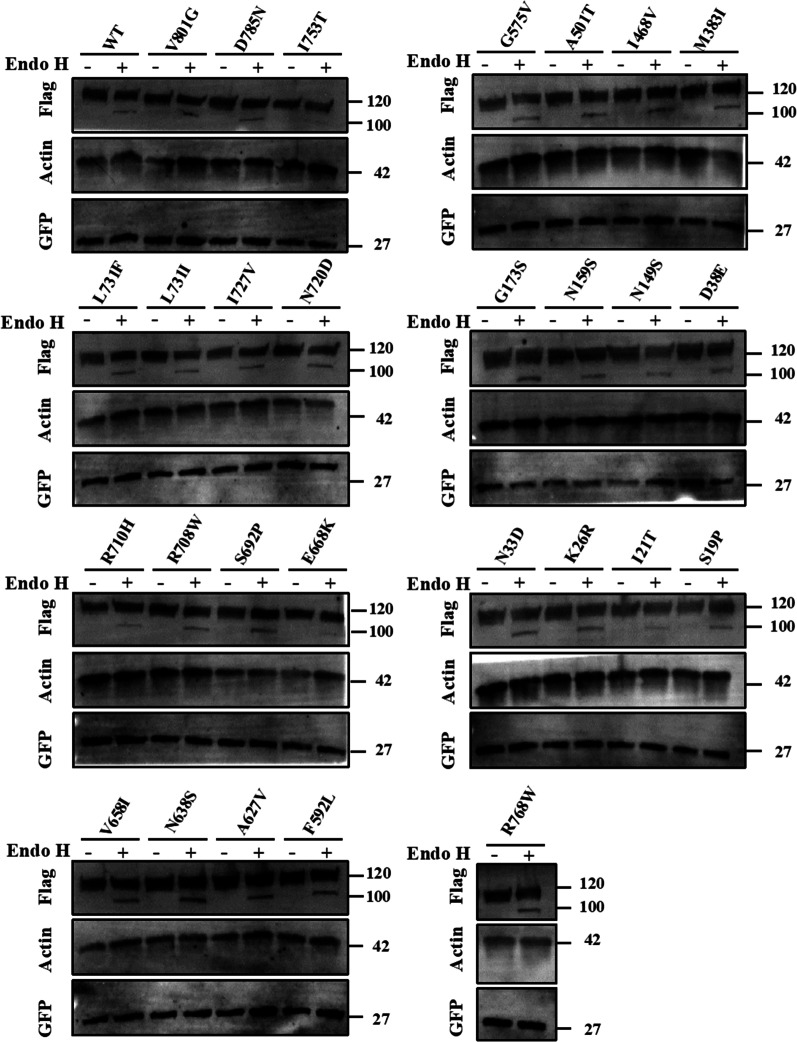
ACE2 variants glycosylation profile. HEK293 cells transiently co-transfected transfected with Flag-tagged ACE2 WT or missense variants and GFP plasmids for 48 Hrs. Lysates were then digested in presence or absence of Endo H enzymes for 3 Hrs. Anti-Flag primary antibody was used to stain WT ACE2 and anti-GFP antibody was used to satin GFP that was used as a transfection control. Actin was used as a loading control. Images were manipulated using ImageJ

To confirm these results, the N- glycosylation profiles of all the studied variants were assessed by Endo H digestion sensitivity and resistance assay and compared to WT ACE2. Similarly, all mutants have shown no significant change in the electrophoretic behaviors compared to WT ACE2, where the vast majority of the expressed ACE2 appears to be resistant to Endo H treatment suggesting that they are fully glycosylated and have normal maturation levels. The Endo H resistant band at ~ 120 KDa accounts for over 96% of the expressed protein for all studied variants. Whereas the lower molecular weight band account to less than ~ 2.8% of total expressed ACE2 as shown in Fig. 5.

## Discussion

The current ongoing COVID-19 pandemic created an urgent need for deciphering the interlink between SARS-CoV-2 and its primary cellular receptor, ACE2. Several studies have extensively investigated ACE2 polymorphic footprint and its associated effects on its structure, binding, and stability [8, 9, 31, 32]. Genetic variations in *ACE2* gene are regarded as a potential risk factor in COVID-19 patients [33]. In this context, different predictive studies based on bioinformatics and simulation tools have generated a bulk of data that helped identify major residues in ACE2 and their consequent effect on SARS-CoV-2 binding [34–36].

In the current study, we have evaluated the impact of several ACE2 coding missense variants using different predictive algorithms and investigated their effect on ACE2 protein subcellular localization, trafficking, and membrane availability. ACE2 gene displays a unique polymorphic profile in the human population in which 332 missense variants were reported in *Ensembl* database. Interestingly, in comparison to its homologue the angiotensin-converting enzyme (ACE), ACE2 displays a lower probability of losing its function by genetic mutations, where probability of being loss-of-function intolerant of ACE2 is pLI = 0.998 by gnomAD database, noting that if pLI > 0.9 gene is considered extremely intolerant [31, 37]. Unlikely, a missense variant in ACE protein (Q1069R) was reported to be associated with renal tubular disease resulting in premature death caused by improper localization of ACE and loss of its function [19]. Although different global or conditioned knockout mouse models have been generated for ACE2, all have reported serious associated phenotypes including developmental, cardiovascular, renal and respiratory clinical manifestations [38].

Overexpressed ACE2 in HEK293 cells in our study was detected at a similar molecular weight ~ 120 KDa compared to endogenous ACE2 detected in other cell lines like HuH7, Caco-2, Calu-3, and HepG2 cells [39]. Moreover, our generated missense variants displayed similar expression profile compared to WT as shown in Additional file 2: Fig. S2. It is worth noting that other ACE2 human variants studied by Shukla et al. and Bhattacharjee et al. (including S19P assessed in our analysis) have displayed no significant change in ACE2 expression level in a similar cellular model [40, 41]. While the surface expression level hasn’t changed, this doesn’t mean that these variants don’t affect the susceptibility of the virus. The binding affinity to the RBD of SARS-CoV-2 was varying significantly compared to WT ACE2 in Bhattacharjee et al. study [41], considering that the affinity-infectivity relationship is still debatable in various studies [42].

All studied genetic variants in this paper had a low population distribution with minor allele frequency less than 1%. Although eight of the variants (K26R, I468V, A627V, N638S, S692P, N720D, and L731F/L731I) were considered major hotspot by Cao et al., and were distributed in different populations, two of them, K26R and N720D, were shown in our analysis to be benign by the different evaluated tools. Stability analysis of K26R and N720D variants was decreased by − 0.34 kcal/mol and − 1.38 kcal/mol (Table 2), respectively. Additionally, glycosylation analysis and immunofluorescence assays display no effect on the cellular trafficking and cell surface localization of these two variants (Additional file 2: Fig S2, Fig. S4 and S5). Substitutions of ACE2 residues involved in SARS-CoV-2 binding have been shown to alter the binding affinity of SARS-CoV-2 S protein [6, 43]. K26 residue is present in the binding domain with SARS-CoV-2 receptor RBD, several studies suggest that residual substitution of lysine with arginine at this site enhances ACE2-SARS-CoV-2 binding and could contribute to higher susceptibility to SARS-CoV-2 [6, 34, 40, 44]. Unlikely, N720 amino acid residue is located in the peptidase domain in a proximity to type II transmembrane serine protease (TMPRSS2) cleavage site. Aside from having lower stable structure compared to WT ACE2, substituting asparagine with aspartic acid at this residue is found to weaken TMPRSS2-ACE2 complex and consequently augmenting SARS-CoV-2 viral entry [31, 45]. Although our analysis displays benign effect of these two variants, and no change in the trafficking and expression profiles, it still can affect their susceptibility to bind to the spike protein. Our analysis has also revealed three deleterious variants (R768W, G575V, and G173S) that were found to affect ACE2 functionality by all the prediction tools in our study. Similarly, intracellular localization and cell surface availability of these variants were not affected in our studied model. Knowing that G575 and G173 are located in the peptidase domain of ACE2 protein, G173S variant is predicted to stabilize ACE2-SARS-CoV-2 complex [46], noting that this substitution might affect ACE2 catalytic activity, while the effect of G575V on the binding affinity to SARS-CoV-2 RBD is not investigated yet. Our results come in line with another in silico analysis showing that R768W is a high risk ACE2 variant and might exert a deleterious effect on ACE2 structure [12]. Additionally, previous studies have shown that the 43 long amino acid topological domain of ACE2 doesn’t affect significantly the cell surface expression and SARS-CoVs mediated viral entry [47, 48]. These data fit with our reported results showing that all mutants present in the cytoplasmic tail of ACE2 (V801G, D785N and R768W) display no change in ACE2 trafficking and transmembrane localization.

In this context, little has been reported in literature about ACE2 biosynthesis and intracellular trafficking. Evident role of N-linked glycosylation on membrane proteins stability, compartmental trafficking, and cell surface expression has been largely reported [49–51]. Among the seven glycosylation sites of ACE2, N90, N322 and N546 form glycan-mediated interactions between ACE2 and SARS-CoV-2 RBD complex [52], noting that N90 and N322 have opposing effects on SARS-CoV-2 binding [30]. ACE2 deprived of all N-glycans was found to accumulate in the endoplasmic reticulum with no significant effect on its enzymatic carboxypeptidase activity [29]. N-linked glycans are usually attached to an asparagine residue of N-X-S/T motif, where X is any amino acid except proline and S/T are serine/threonine amino acids. Among the variants we were interested in studying is S692P, two amino acids proximal to N690 glycosylation site. Substituting serine for proline in the S/T site would consequently lead to the loss of N-glycosylation at N690. We show in our analysis that this substitution increases ACE2 stability (ΔΔG = 1.41 kcal/mol), however, immunofluorescence data show no change in the trafficking of this variant compared to the WT ACE2.

## Conclusion

In summary, throughout this study, we show that none of the coding variants included in our work display an effect on ACE2 protein subcellular trafficking and its cell surface availability. This might be due to the high importance of this gene and therefore its intolerance to the loss of its function. Noting that the deleterious effect reported by the computational tools might be affecting the carboxypeptidase activity and binding affinity of ACE2 that were not evaluated in our paper, rather than its trafficking and SARS-CoVs related effect. Apart from ACE2 trafficking modulation, the receptor membranous expression and availability might be influenced at different levels [53]. Considering that ACE2 gene lies on the X chromosome, it is strongly evident that females have an advantage due to the greater chance to form structurally variable ACE2 dimers compared to males who are hemizygous for the ACE2 gene [54]. ACE2 expression is highly affected by sex hormones where estrogen strongly elevates its expression explaining the variable effect of COVID-19 in both sexes [55]. Moreover, ACE2 expression might also be affected by different epigenetic changes providing some evidence to explain the difference in the interindividual susceptibility and clinical variability in infected patients [56, 57]. It is worth noting that a recent Mendelian Randomization (MR) study suggests that ACE2 expression is a causative player in the susceptibility and severity of SARS-CoV-2 infection [58]. Although ACE2 genetic variants didn’t display any significant effect on ACE2 cell surface availability through its trafficking pathway, it still might affect the pharmacokinetics of ACE2 including its decay and consequently SARS-CoV-2 entry. Collectively, these data have important implication on COVID-19 progression and to face these outcomes more detailed knowledge is needed to understand the normal mechanisms controlling ACE2 expression and trafficking at the cellular level as well as its role in the pathogenesis of SARS-CoVs infection.

## Supplementary Information

**Additional file 1.** list of ACE2 mutagenesis primers and benign ACE2 variants by prediction tools.

**Additional file 2.** Supplementary figures.

## Data Availability

All data generated or analyzed during the study are included in this article and its additional files. Any further requirements are available from the corresponding author upon request.

## Abbreviations

3D: Three-dimensional
AA: Amino acid
ACE: Angiotensin-converting enzyme
ACE2: Angiotensin-converting enzyme 2
Ang II: Angiotensin II
CHX: Cycloheximide
COVID-19: Corona Virus Disease-2019
DMSO: Dimethyl sulfoxide
Endo H: Endoglycosidase H
ER: Endoplasmic reticulum
ERAD: Endoplasmic reticulum-associated degradation
Hrs: Hours
PBS: Phosphate-buffered serum
PD: Peptidase domain
pLI: Probability of being loss-of-function intolerant
PNGase F: Peptide N-glycosidase F
RBD: Receptor binding domain
SARS-CoVs: Severe acute respiratory syndrome corona viruses
SNP: Single nucleotide polymorphism
SP: Signal peptide
TMPRSS2: Type II transmembrane serine protease
WT: Wild type
