# Supplementary material for: Characterization of ACE2 naturally occurring missense variants: impact on subcellular localization and trafficking
Source: Hum Genomics. 2022 Sep 2;16:35. doi: 10.1186/s40246-022-00411-1 (PMC9438391; doi:10.1186/s40246-022-00411-1)
Supplement: Supplementary file 1 — Additional file 1. list of ACE2 mutagenesis primers and benign ACE2 variants by prediction tools. [file 40246_2022_411_MOESM1_ESM.docx]

**Table S1**: ACE2 primers used to generate missense mutants

| **ACE2 mutant** | **Forward Primer** | **Reverse Primer** |
| --- | --- | --- |
| p.Val801Gly  c.2402T>G | 5' ACTGATGATGGTCAGACCTCCTT 3' | 5' AAGGAGGTCTGACCATCATCAGT 3' |
| p.Asp785Asn  c.2353G>A | 5' TGCCTCCATCAATATTAGCAAAGG 3' | 5' CCTTTGCTAATATTGATGGAGGCA 3' |
| p.Arg768Trp  c.2302C>T | 5' GGGATCAGAGATTGGAAGAAGAAAA 3' | 5' TTTTCTTCTTCCAATCTCTGATCCC 3' |
| p.Ile753Thr  c.2258T>C | 5' ATGGGAGTGACAGTGGTTGGCA 3' | 5' TGCCAACCACTGTCACTCCCAT 3' |
| p.Leu731Phe  c.2191C>T | 5' TACAGCCAACAATTGGACCTCCT 3' | 5' AGGAGGTCCAATTGTTGGCTGTA 3' |
| p.Leu731Ile  c.2191C>A | 5' TACAGCCAACATTTGGACCTCCT 3' | 5' AGGAGGTCCAAATGTTGGCTGTA 3' |
| p.Ile727Val  c.2179A>G | 5' AGTTTCTGGGGGTACAGCCAAC 3' | 5' GTTGGCTGTACCCCCAGAAACT 3' |
| p.Asn720Asp  c.2158A>G | 5' GTCTGAATGACGACAGCCTAGA 3' | 5' TCTAGGCTGTCGTCATTCAGAC 3' |
| p.Arg710His  c.2129G>A | 5' GTCCCGGAGCCATATCAATGAT 3' | 5' ATCATTGATATGGCTCCGGGAC 3' |
| p.Arg708Trp  c.2122C>T | 5' CAGGATGTCCTGGAGCCGTAT 3' | 5' ATACGGCTCCAGGACATCCTG 3' |
| p.Ser692Pro  c.2074T>C | 5' CCTAAAAATGTGCCTGATATCATTCC 3' | 5' GGAATGATATCAGGCACATTTTTAGG 3' |
| p.Glu668Lys  c.2002G>A | 5' TTTTTGGGGAGAAGGATGTGCG 3' | 5' CGCACATCCTTCTCCCCAAAAA 3' |
| p.Val658Ile  c.1972G>A | 5'GGCAGTACTTTTTAAAAATAAAAAATCAGAT3' | 5'CATCTGATTTTTTATTTTTAAAAAGTACTGCC 3' |
| p.Asn638Ser  c.1913A>G | 5' TGGAACGACAGTGAAATGTACC 3' | 5' GGTACATTTCACTGTCGTTCCA 3' |
| p.Ala627Val  c.1880C>T | 5' GCCTAAAATCAGTTCTTGGAGAT 3' | 5' ATCTCCAAGAACTGATTTTAGGC 3' |
| p.Phe592Leu  c.1774T>C | 5' TTGAGCCCTTACTTACCTGGCT 3' | 5' AGCCAGGTAAGTAAGGGCTCAA 3' |
| p.Gly575Val  c.1724G>T | 5' GGAAAATGTTGTAGTAGCAAAGAACATG 3' | 5' CATGTTCTTTGCTACTACAACATTTTCC 3' |
| p.Ala501Thr  c.1501G>A | 5' ACTGTGACCCCACATCTCTGTT 3' | 5' AACAGAGATGTGGGGTCACAGT 3' |
| p.Ile468Val  c.1402A>G | 5' TAAAGGGGAAGTTCCCAAAGAC 3' | 5' GTCTTTGGGAACTTCCCCTTTA 3' |
| p.Met383Ile  c.1149G>A | 5' CCAGTATGATATAGCATATGCTGC 3' | 5' GCAGCATATGCTATATCATACTGG 3' |
| p.Gly173Ser  c.517G>A | 5' ATCTGAGGTCAGCAAGCAGCT 3' | 5' AGCTGCTTGCTGACCTCAGAT 3' |
| p.Asn159Se  c.476A>G | 5' AGTTTAGACTACAGTGAGAGGCTCT 3' | 5' AGAGCCTCTCACTGTAGTCTAAACT 3' |
| p.Asn149Se  c.446A>G | 5' AACCAGGTTTGAGTGAAATAATGGC 3' | 5' GCCATTATTTCACTCAAACCTGGTT 3' |
| p.Asp38Glu  c.114C>G | 5' GAAGCCGAAGAGCTGTTCTATCAAA 3' | 5' TTTGATAGAACAGCTCTTCGGCTTC 3' |
| p.Asn33Asp  c.97A>G | 5' TTTGGACAAGTTTGACCACGAAGCC 3' | 5' GGCTTCGTGGTCAAACTTGTCCAAA 3' |
| p.Lys26Arg  c.77A>G | 5' AGGAACAGGCCAGGACATTTTTGGA 3' | 5' TCCAAAAATGTCCTGGCCTGTTCCT 3' |
| p.Ile21Thr  c.62T>C | 5' CAGTCCACCACTGAGGAACAG 3' | 5' CTGTTCCTCAGTGGTGGACTG 3' |
| p.Ser19Pro  c.55T>C | 5' TGCTGCTCAGCCCACCATTGA 3' | 5' TCAATGGTGGGCTGAGCAGCA 3' |

**Table S2:** ACE2 benign missense variants by the different prediction tools.

|  |  |  |  | **SIFT** | | **PROVEAN** | | **HumanDiv PolyPhen.2** | | **HumanVar PolyPhen.2** | | **Mutation taster** |
| --- | --- | --- | --- | --- | --- | --- | --- | --- | --- | --- | --- | --- |
| **ACE2 mutant** | **SNP** | **AA substitution** | **genomAD allele frequency** | **Effect** | **Score** | **Prediction** | **Score** | **Prediction** | **Score** | **Prediction** | **Score** | **Prediction** |
| c.2402T>G | rs1464340051 | V801G | 5.617e-06 | Tol | 0.18 | Neu | -0.730 | Ben | 0.001 | Ben | 0.003 | Pol |
| c.2158A>G | rs41303171 | N720D | 0.016 | Tol | 0.10 | Neu | -1.192 | Ben | 0.009 | Ben | 0.041 | Pol |
| c.2002G>A | rs200180615 | E668K | 2.2e-05 | Tol | 0.27 | Neu | -0.547 | Ben | 0.002 | Ben | 0.004 | Pol |
| c.1972G>A | rs1295899858 | V658I | 5.956e-06 | Tol | 0.52 | Neu | -0.187 | Ben | 0 | Ben | 0.001 | Pol |
| c.1774T>C | NA | F592L | NA | Tol | 0.64 | Neu | 0.133 | Ben | 0.017 | Ben | 0.017 | Pol |
| c.476A>G | rs746034076 | N159S | 1.637e-05 | Tol | 0.49 | Neu | -0.738 | Ben | 0.004 | Ben | 0.005 | Pol |
| c.446A>G | rs373252182 | N149S | NA | Tol | 0.32 | Neu | -0.036 | Ben | 0.013 | Ben | 0.033 | Pol |
| c.114C>G | NA | D38E | NA | Tol | 1.00 | Neu | -0.327 | Ben | 0 | Ben | 0.001 | Pol |
| c.77A>G | rs4646116 | K26R | 0.004 | Tol | 0.45 | Neu | -0.579 | Ben | 0 | Ben | 0.001 | Pol |
| c.62T>C | rs1244687367 | I21T | 5.463e-06 | Tol | 0.80 | Neu | 1.123 | Ben | 0.001 | Ben | 0.03 | Pol |

Abbreviation: Tol: Tolerated, Neu: Neutral, Ben: Benign, Pol: Polymorphism, NA: Not available.

**Supplementary figures’ legends**

**Fig S1.** ACE2 expression and localization in HEK293 and HeLa cell lines.

(A) HEK293 and HeLa cell lysates were subjected to SDS-PAGE assay where the membrane was stained with specific anti-ACE2 antibody and its corresponding secondary antibody to check its endogenous expression. ACE2-transfected HEK293 cell lysates were included in the blot as a positive control. Actin was used as loading control. (B) HEK293 and HeLa cells were co-transfected with Flag-tagged ACE2 and GFP-tagged HRas (plasma membrane marker in green) plasmids for immunofluorescence. Cells were fixed and stained with anti-Flag primary and its corresponding secondary (Red) antibodies. Images were acquired using 100X magnification and manipulated by ImageJ. Scale bar = 50 μm.

**Fig S2.** Wild type and mutated ACE2 overexpression

HEK293 cells transiently co-transfected with Flag-tagged ACE2 WT or missense variants and GFP plasmids. 48 Hrs post-transfection, cells were harvested and subjected to SDS-PAGE analysis for ACE2 expression. Membranes were blotted with anti-Flag and anti-GFP primary and their corresponding secondary antibodies at their distinctive molecular weights. Actin was used as loading control in all presented blots. Images were manipulated using ImageJ.

**Fig S3.** ACE2 benign variants subcellular localization

HeLa cells were grown on coverslips and transiently co-transfected with Flag-tagged ACE2 WT or missense variants and GFP-tagged HRas (plasma membrane marker). 24 Hrs post transfection, cells were fixed and stained with anti-Flag and anti-Calnexin (ER marker) antibodies. Images were acquired using 100X magnification and manipulated by ImageJ. Scale bar = 50 μm.
