# Supplementary figures and images for: Characterization of ACE2 naturally occurring missense variants: impact on subcellular localization and trafficking
Source: Hum Genomics. 2022 Sep 2;16:35. doi: 10.1186/s40246-022-00411-1 (PMC9438391; doi:10.1186/s40246-022-00411-1)

Fig. S1

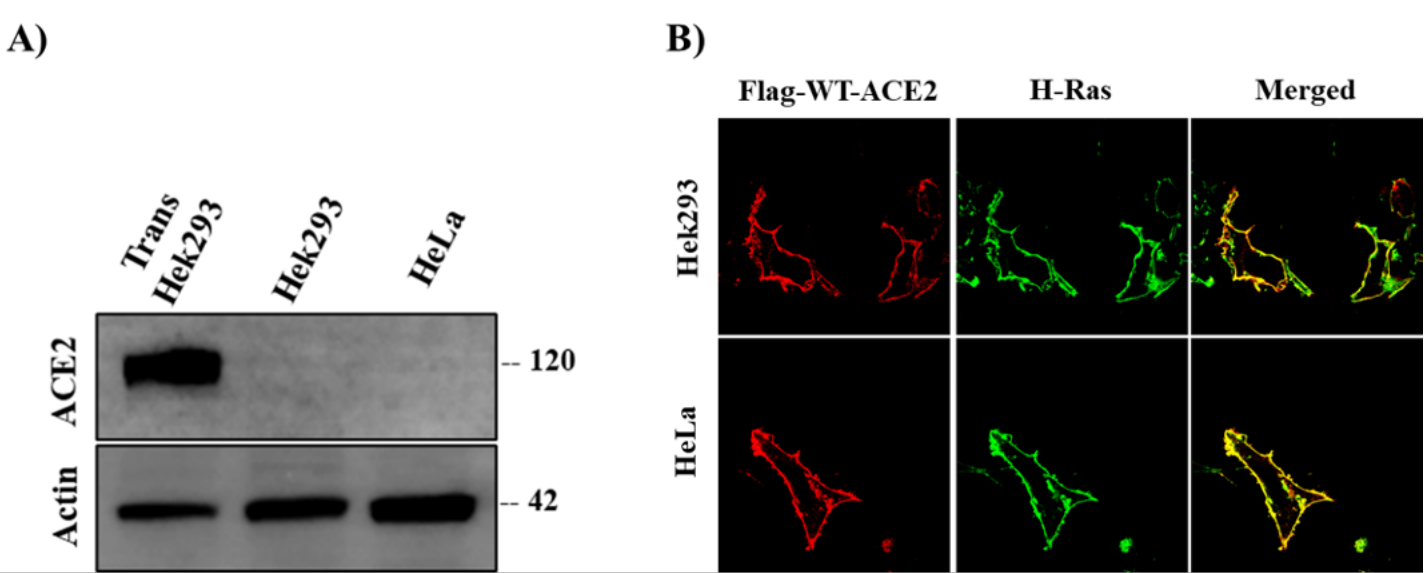

Fig. S2

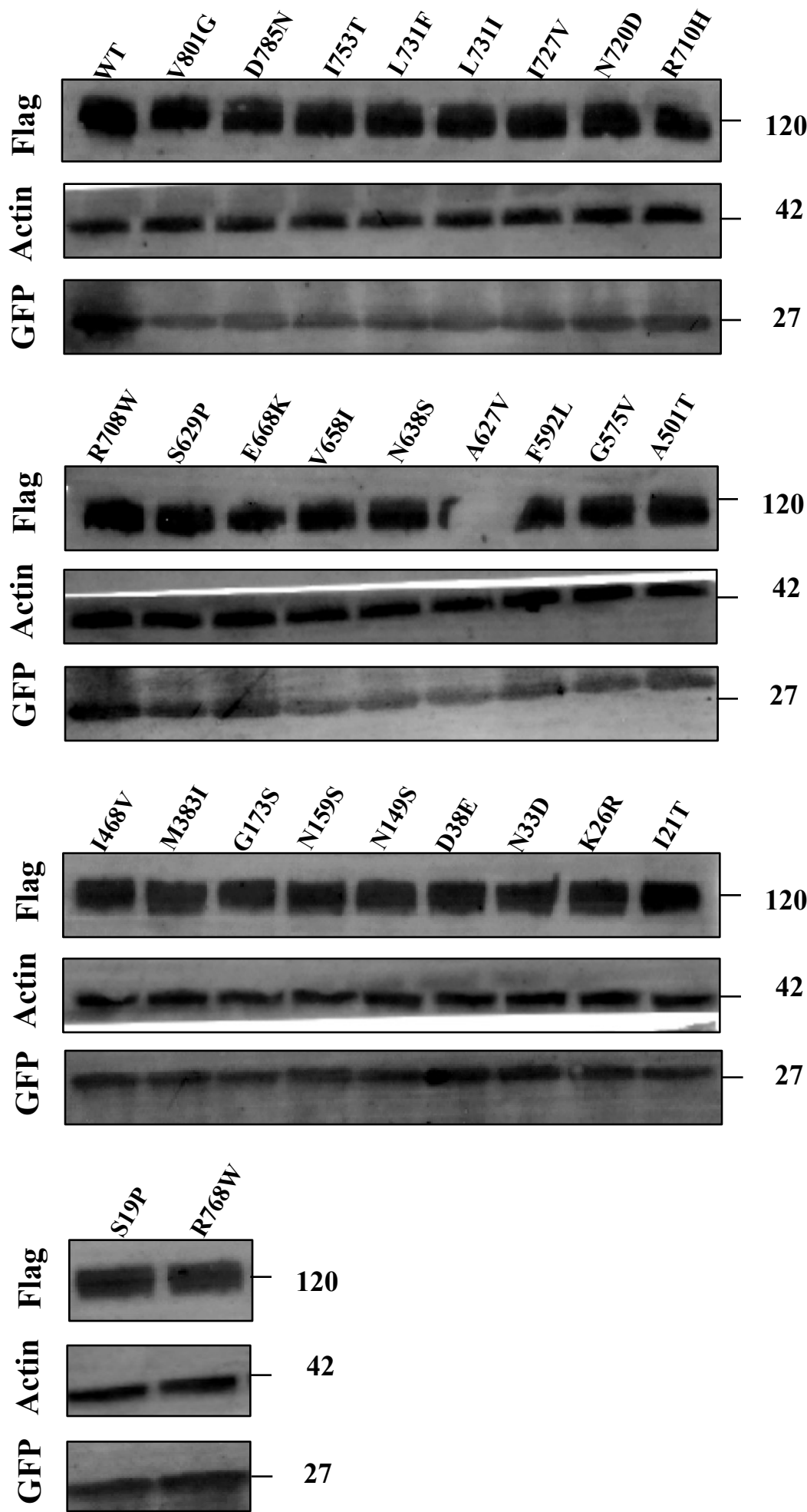

Fig. S3

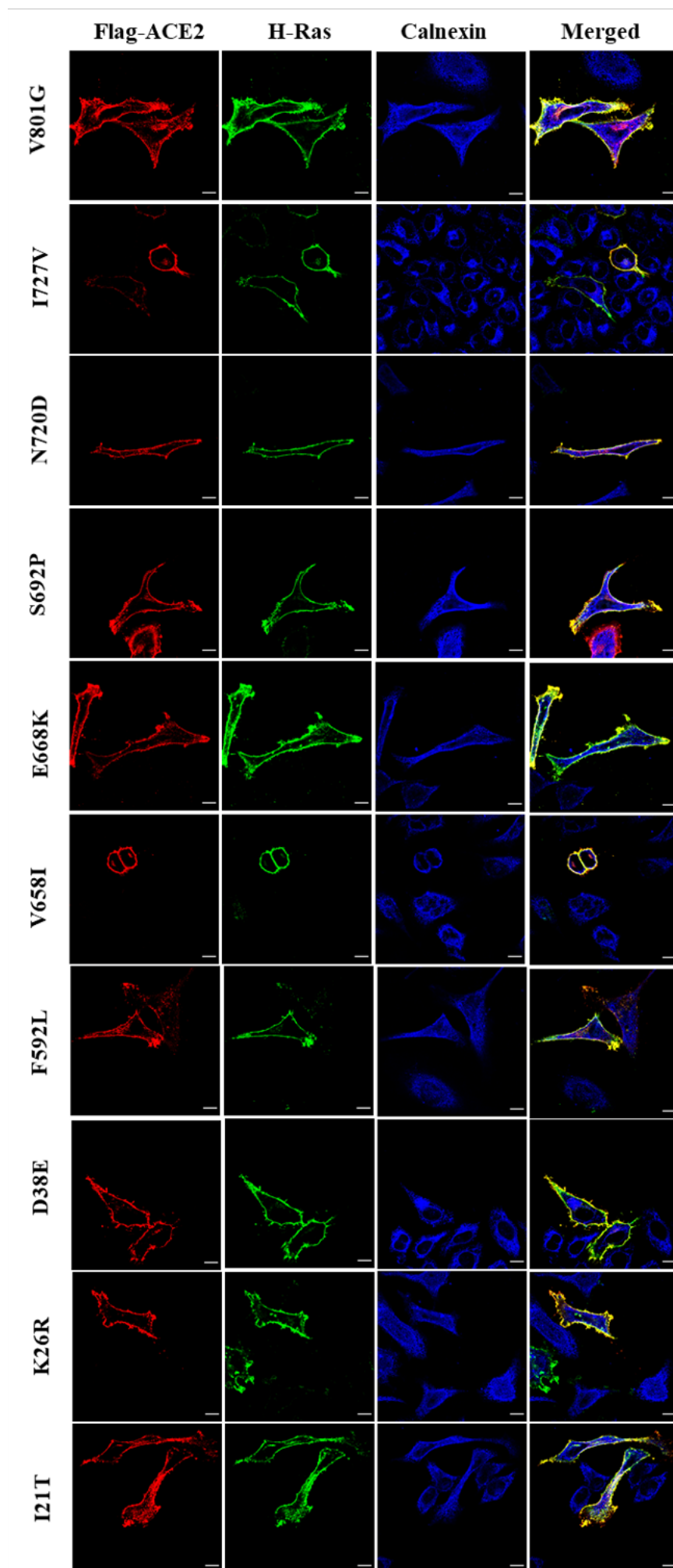

Supplement: Supplementary file 2 — Additional file 2. Supplementary figures. [file 40246_2022_411_MOESM2_ESM.pdf]
